# Evaluation of urinary volatile organic compounds as a novel metabolomic biomarker to assess chronic kidney disease progression

**DOI:** 10.1186/s12882-024-03819-0

**Published:** 2024-10-15

**Authors:** Henry H. L. Wu, Malcolm Possell, Long The Nguyen, Wenbo Peng, Carol A. Pollock, Sonia Saad

**Affiliations:** 1 Renal Research Laboratory, Kolling Institute of Medical Research, Royal North Shore Hospital & The University of Sydney, Sydney, Australia; 2 Centre for Carbon, Water and Food, School of Life and Environmental Sciences, The University of Sydney, Sydney, Australia; 3 School of Public Health, Faculty of Health, University of Technology Sydney, Sydney, Australia; 4 Department of Renal Medicine, Royal North Shore Hospital, Northern Sydney Local Health District, Sydney, Australia

**Keywords:** Chronic kidney disease, Volatile organic compounds, Translational diagnostics, Interstitial fibrosis and tubular atrophy, Non-invasive diagnosis

## Abstract

**Background:**

There is a need to develop accurate and reliable non-invasive methods to evaluate chronic kidney disease (CKD) status and assess disease progression. Given it is recognized that dysregulation in metabolic pathways occur from early CKD, there is a basis in utilizing metabolomic biomarkers to monitor CKD progression. Volatile Organic Compounds (VOCs), a form of metabolomic biomarker, are gaseous products of metabolic processes in organisms which are typically released with greater abundance in disease conditions when there is dysregulation in metabolism. How urinary VOCs reflect the abnormal metabolic profile of patients with CKD status is unknown. Our study aimed to explore this.

**Methods:**

Individuals aged 18–75 years undergoing kidney biopsy were included. Pre-biopsy urine samples were collected. All biopsy samples had an interstitial fibrosis and tubular atrophy (IFTA) grade scored by standardized assessment. Urine supernatant was extracted from residue and sampled for stir bar sorptive extraction followed by Gas chromatography–mass spectrometry (GC-MS) analysis. Post-processing of GC-MS data separated complex mixtures of VOCs based on their volatility and polarity. Mass-to-charge ratios and fragment patterns were measured for individual VOCs identification and quantification. Linear discriminant analysis (LDA) was performed to assess the ability of urinary VOCs in discriminating between IFTA 0 (‘no or minimal IFTA’ i.e. <10%, IFTA), IFTA 1 (‘mild IFTA’ i.e. 10–25% IFTA) and IFTA ≥ 2 (‘moderate or severe IFTA’ i.e. >25% IFTA). Linear regression analysis adjusting for age, sex, estimated glomerular filtration rate, diabetes mellitus (DM) status, and albuminuria was conducted to determine significantly regulated urinary VOCs amongst the groups.

**Results:**

64 study participants (22 individuals IFTA 0, 15 individuals IFTA 1, 27 individuals IFTA ≥ 2) were included. There were 34 VOCs identified from GC-MS which were statistically associated with correct classification between the IFTA groups, and LDA demonstrated individuals with IFTA 0, IFTA 1 and IFTA ≥ 2 could be significantly separated by their urinary VOCs profile (*p* < 0.001). Multivariate linear regression analysis reported 4 VOCs significantly upregulated in the IFTA 1 compared to the IFTA 0 group, and 2 VOCs significantly upregulated in the IFTA ≥ 2 compared to the IFTA 1 group (*p* < 0.05). Significantly upregulated urinary VOCs belonged to one of four functional groups - aldehydes, ketones, hydrocarbons, or alcohols.

**Conclusions:**

We report novel links between urinary VOCs and tubulointerstitial histopathology. Our findings suggest the application of urinary VOCs as a metabolomic biomarker may have a useful clinical role to non-invasively assess CKD status during disease progression.

## Background

An increase in life expectancy and an increasing prevalence of diabetes mellitus and obesity has amounted to a greater number of individuals affected by chronic kidney disease (CKD), with more than 10% of the global population affected by this condition currently [1]. By 2040, CKD is projected to emerge as the fifth-leading cause of mortality worldwide [2]. Early diagnosis is important to allow for timely intervention which may reduce the excess morbidity and mortality in patients with CKD.

Histopathological evaluation of kidney biopsy tissue remains the gold standard approach which accurately reflects any presence of kidney pathology. Serially performing kidney biopsies to monitor kidney status is not ideal however, as it is invasive and costly. Traditional serum and urine-based tests such as estimated glomerular filtration rate (eGFR) and urinary albumin, whilst considered convenient routinely performed tests to determine kidney function, do have limitations when aiming to accurately assess kidney disease status [3–5]. There remains a need to develop reliable non-invasive methods to evaluate kidney disease status. To this end, the emergence of novel proteomic and metabolomic techniques to determine specific biomarkers which inform on the metabolic and kidney disease status of an individual has taken significant strides [6–8].

Utilization of volatile organic compounds (VOCs) as non-invasive metabolomic biomarkers to evaluate metabolic and kidney status has received growing interest over recent years [9–12]. VOCs are gaseous products of metabolic processes in organisms which are conventionally released with greater abundance in disease conditions when there is dysregulation in metabolism [13]. Due to the kidneys’ extraction of soluble wastes from the bloodstream and pre-concentration capabilities, urine has considerable value as a source of VOCs which may reflect the state and function of the kidneys, as well as other organs and pathologies. More than 400 human urinary VOCs – ranging across different organic chemistry functional groups (e.g. alcohols, benzenes, ketones, hydrocarbons, pyrroles, furans, aldehydes, terpenes, sulfur-containing compounds (isocyanates, sulfides), and O- and N-heterocyclic compounds – have been previously identified in normal physiological conditions and in various pathological conditions [14]. Whether the expression levels of VOCs in human urine can play a considerable role in accurately assessing CKD status remains unknown to date. Our study aimed to evaluate whether urinary expression levels of VOCs are significantly associated with the degree of tubulointerstitial fibrosis in the kidney, as reported by kidney biopsy.

## Methods

### Study participant recruitment and ethical considerations

Adult individuals of either sex aged between 18 and 75 years of age under the care of the Department of Renal Medicine at Royal North Shore Hospital or North Shore Private Hospital, Sydney, Australia referred for kidney biopsy were included in this study. Individuals receiving kidney replacement therapy were excluded from this study. Informed consent was obtained from all study participants. Data collection in this study was carried out in accordance with relevant local guidelines and regulations, and collection of human data was approved by the human ethics committee at Royal North Shore Hospital (Ref: HREC/17/HAWKE/471).

### Evaluation of kidney biopsy tissue for interstitial fibrosis and tubular atrophy grading to determine study participant groups

The procurement of kidney biopsy tissue was performed in the Medical Day Procedure Unit at Royal North Shore Hospital. Prior to commencing the procedure, written consent was obtained from study participants to collect the pre-biopsy urine sample for purposes of this study, and to obtain access to the kidney biopsy tissue, which was otherwise performed for clinical indications. Tissue obtained from kidney biopsies were subsequently transferred to the histopathology department and assessed as per standard protocols to determine interstitial fibrosis and tubular atrophy (IFTA) grading. Kidney biopsy samples were processed for light microscopic evaluation via paraffin-embedded sections, supplemented by special and immune histochemical (IHC) stains. Some samples were reserved for immunofluorescence and electron microscopic studies if indicated. Light microscopy assessment included a minimum of two hematoxylin and eosin (H&E), two periodic acid-Schiff (PAS), two Masson’s trichrome (trichrome), and two Jones methenamine silver (silver) stains in complementary fashion. H&E stains provided a general overview of all structures, cytoplasmic and nuclear features, PAS stains highlighted tubular and glomerular basement membranes, trichrome stains accentuated fibrous tissue and fibrin, if present, and silver stains highlighted the glomerular and tubular basement membranes, and also sclerosis. The biopsy assessment was conducted blindly by three accreditated pathologists from the NSW Health Pathology Laboratory, Department of Anatomical Pathology, Northern Sydney Local Health District, Sydney, Australia. Kidney biopsy tissue was assessed as having IFTA 0 (‘no or minimal IFTA’ i.e. <10%, IFTA), IFTA 1 (‘mild IFTA’ i.e. 10–25% IFTA) and IFTA ≥ 2 (‘moderate or severe IFTA’ i.e. >25% IFTA).

Study participants’ demographic alongside clinical and biochemical data were acquired from the Royal North Shore Hospital PowerChart Database, summarized using appropriate descriptive statistics and compared between the three groups. For demographic and clinical variables with symmetric normal distributions, the mean and standard deviation were reported. For variables that were skewed or ordinal, the median and interquartile range were used for statistical purposes. Proportions were also presented for categorical variables. Continuous variables between the groups were compared using the Analysis of Variance (ANOVA) test (if normally distributed) or Kruskal-Wallis test (if the distribution was non-parametric). Categorical variables were compared using the Chi-square test or Freeman-Halton extension of the Fisher’s exact test accounting for sparsely distributed data.

### Collection of urine samples and transferring sample for stir bar sorptive extraction

Using urine bottles with capacity of up to 100 ml, spot urine samples were collected from adult individuals who fulfilled the study criteria. Each collected urine sample was placed on ice immediately after collection for transportation to the Renal Research Laboratory, Kolling Institute of Medical Research and were centrifuged for 20 min at 4℃ to isolate urine supernatant from residue. Urine supernatant were stored at -80 °C and defrosted overnight at 4 °C before further sampling. 5 ml of urine was transferred to a 20 ml headspace vial and 3 µl of 15ppm bromobenzene in methanol internal standard (IS) was added along with a conditioned polydimethylsiloxane phase stir bar (Twister, 10 mm x 0.5 mm film thickness; Gerstel, Mülheim an der Ruhr, Germany). The headspace vial was capped and Stir Bar Sorptive Extraction (SBSE) proceeded, with the stir bar spun at 800 rpm for 2 h. The stir bar was then removed, rinsed with double distilled water and patted dry with a lint free tissue before analysis. Two blank samples consisting of 5 ml of double-distilled water, spiked with the same IS were run with each cohort of samples. To determine retention indices, 1 µl of C8-C20 homologous n-alkanes (containing approximately 40 mg/l of each alkane) was injected onto separate, conditioned SBSE. All reagents were sourced from Sigma-Aldrich (Sydney, Australia).

### Gas chromatography–mass spectrometry analysis

Thermal desorption (TD) of the stir bars was done using a Gerstel Thermal Desorption Unit (TDU; Gerstel, Mülheim an der Ruhr, Germany). SBSE stir bars were placed into glass thermal desorption liners that were inserted into the TDU for analysis. Upon insertion into the TDU, the samples were purged with ultra-high purity helium (BOC Ltd, North Ryde, NSW, Australia) at 35 °C for 1 min to eliminate air from the sample and inlet. Samples were then heated by the TDU at 12 °C/s to 250 °C with a helium flow of 50 ml/min. TD products were carried by the helium through to a programmed temperature vaporization (PTV) inlet (CIS-4; Gerstel) installed in an Agilent 7890GC (Agilent Technologies Pty Ltd, Mulgrave, Australia), which was used in solvent mode during the TD. The PTV inlet, containing a glass liner filled with Tenax TA, was held at 30 °C during the TD using liquid CO_2_ (BOC Ltd) as the cryogen. After 5 min of TD, the CIS-4 was heated at 12 °C/s to 250 °C and held at that temperature for 3 min while the TD products were injected into the GC without splitting. TD products were separated on a HP-5ms capillary column (30 m x 0.25 mm, 0.25 μm film thickness; Agilent), which was connected to a mass selective detector (Model 5975 C; Agilent). Ultra-high purity helium was used as carrier gas (flow rate through the HP-5ms column was 2.3 ml/min). The initial oven temperature of the GC was 40 °C, held for 2 min, then heated at a rate of 4 °C/min to 250 °C and held for 5 min. The temperature of the Gas chromatography-mass spectrometry (GC-MS) interface was 280 °C, the MS ion source 230 °C and the quadrupole 150 °C. The detector, in electron impact mode (70 eV), scanned the range of 35–300 m/z. Operation of the GC-MS was controlled by Agilent Chemstation (version E.02.01.117) and the TDU by Maestro (version 1.4.36.16; Gerstel).

### Quality Control

Pooled urine quality control (QC) samples were generated for each of the three cohorts (IFTA 0; IFTA 1; IFTA ≥ 2) by mixing an equal volume of urine of each study sample to make a total of 30 ml of urine. This allowed for 6 × 5 ml QC samples for each cohort. These were extracted and analyzed as described for the study samples.

### Gas chromatography–mass spectrometry post processing of urinary volatile organic compound data

Post-processing of GC-MS data to separate complex mixtures of VOCs based on their volatility and polarity, and measuring mass-to-charge ratios and fragment patterns for individual VOC identification and quantification was performed. Chromatograms were batch processed by metaMS (version 2.1.1) [15], hosted on the Workflow4Metabolomics Galaxy Server [16]. metaMS outputs a data matrix of aligned mass spectra with their corresponding peak area and a mass spectral pattern file. The maximum peak area of aligned mass spectra of the two water blanks run in every batch of samples were subtracted before further analysis. Mass spectra were considered reproducible if they were present in four out of six QC samples, the presence in the QC samples had a coefficient of variation < 30% and the dispersion ratio (a measure of variance in the QC samples to those of the urine samples) was less than 50% [17]. The mass spectra were identified against the NIST14 mass spectral library in NIST MS Search (NIST MS Search v.2.2; NIST, Gaithersburg, MD) using a match factor threshold of 700, and closeness to available retention index value (using non-isothermal Kovats’ Retention Indices from the definition of van den Dool and Kratz, for a semi-standard non-polar column) [18].

### Statistical analysis of post-processed urinary volatile organic compound data

Expression levels of identified VOCs were compared across the 3 study participant groups. To determine the importance of VOCs and their presence to differentiate IFTA status, linear discriminant analysis (LDA), a supervised learning technique, was used to distinguish the groups. The Mahalanobis distance between each group was calculated to validate the LDA model. Leave-one-out (LOO) cross validation was performed to determine the classification correctness rate of the VOCs across the 3 IFTA groups.

A number of statistical methods were used, including descriptive statistics, one-way ANOVA with *post hoc* Bonferroni correction, and Kruskal–Wallis test according to the data types and distributions. Associations between the expression levels of identified urinary VOCs and IFTA grading were then evaluated by linear regression analyses. Linear relationships between the dependent and independent variables, multivariate normality (via Q-Q plots of the residuals), and multicollinearity were checked before implementing the regression models. For eligible VOCs, two linear regression models were performed – the univariate model and a multivariate model adjusting for age, sex, estimated glomerular filtration rate (eGFR), diabetes mellitus (DM) status (i.e. no DM or DM), and albuminuria (i.e. no albuminuria, microalbuminuria or macroalbuminuria) of study participants. Covariates were selected a priori. In the multivariate model, a secondary analysis evaluating between the expression levels of identified urinary VOCs and covariates was also completed. Coefficient values, standard error (SE) values and 95% confidence intervals (95%CI) were reported for each model. All statistical tests were 2-sided, and *p* < 0.05 was considered statistically significant. Statistical analyses were performed using Stata 16 (StataCorp MP, College Station, TX, USA).

## Results

### Characteristics of study participants

The relevant demographic, clinical and biochemistry characteristics of study participants are presented in Table 1. The three study groups included 22 individuals diagnosed with IFTA 0, 15 individuals diagnosed with IFTA 1, and 27 individuals diagnosed with IFTA ≥ 2 upon evaluation of kidney biopsy. There were statistically significant differences in age, level of eGFR and albuminuria among the three groups (both *p* < 0.05), while sex and the presence of diabetes displayed no statistically significant differences between the three groups. As such, study participants with more severe IFTA were older, had lower eGFR and more severe albuminuria, as expected, compared to the other two groups.

**Table 1:** Relevant characteristics of the study participants by IFTA status (*n* = 64)

	All participants	IFTA 0 (*n* = 22)	IFTA 1 (*n* = 15)	IFTA ≥ 2 (*n* = 27)	*p*-value*
**Age in years**, mean (SD)	46 (16)	38 (13)	50 (14)	51 (17)	**0.007**
**Sex in n (%)**					0.771
Female	31 (48%)	12 (39%)	7 (22%)	12 (39%)	
Male	33 (52%)	10 (30%)	8 (24%)	15 (46%)	
**eGFR in ml/min/1.73m**^**2**^, mean (SD)	65 (26)	90 (0)	71 (18)	40 (15)	**<0.001**
**Diabetes in n (%)**					0.113
With diabetes	6 (9%)	0	3 (50%)	3 (50%)	
Without diabetes	58 (91%)	22 (38%)	12 (21%)	24 (41%)	
**Albuminuria in n (%)^**					
No albuminuria	29 (45%)	22 (100%)	2 (13%)	5 (19%)	**<0.001**
Microalbuminuria	18 (28%)	0 (0%)	4 (27%)	14 (52%)	
Macroalbuminuria	17 (27%)	0 (0%)	9 (60%)	8 (29%)	

eGFR: Estimated glomerular filtration rate; IFTA: Interstitial fibrosis and tubular atrophy; SD: Standard deviation

*p-values were adjusted by Bonferroni’s correction

### Characteristics of post-processed urinary volatile organic compound data

There were 34 urinary VOCs which were identified following GC-MS post-processing. A summary of the expression levels in relation to each identified urinary VOC across the three IFTA groups is described in Table 2. The expression levels of 29 urinary VOCs have appeared with a ‘zero’ value in one or two IFTA groups, and 5 urinary VOCs had mean values different from a ‘zero’ value for all three IFTA groups. These 5 urinary VOCs are Benzeneacetaldehyde, α-methyl-; Benzaldehyde, 4-propyl-; Phenol, 2,5-bis(1,1-dimethylethyl)-; Hexamethylene diacrylate; and 2(3 H)-Furanone, dihydro-5-(2-octenyl)-, (Z)-. Amongst these 5 urinary VOCs, there were statistically significant differences in the Phenol, 2,5-bis(1,1-dimethylethyl)- levels between the three IFTA groups. Compared to study participants with IFTA 0, those with IFTA 1 and IFTA ≥ 2 had statistically significantly higher Phenol, 2,5-bis(1,1-dimethylethyl)- levels. The Phenol, 2,5-bis(1,1-dimethylethyl)- level among people with IFTA ≥ 2 was significantly higher than those with IFTA 1 (all *p* < 0.05).

**Table 2:** Characteristics of post-processed urinary volatile organic compound data by IFTA status (GC-MS peak area; *n* = 34)

Compound	IFTA 0 (*n* = 22)	IFTA 1 (*n* = 15)	IFTA ≥ 2 (*n* = 27)	*p*-value*
	Mean (SD)	Mean (SD)	Mean (SD)	
2,3-Butanedione	0	0	255,635 (484396)	**<0.001**
m/p-xylene	0	31,079(28600)	40,663 (102840)	**<0.001**
4-Heptanone	869,378 (804062)	487,512 (900704)	0	**<0.001**
Styrene	0	53,015 (41151)	132,887 (320523)	**<0.001**
2-Heptanone	0	25,230 (40867)	0	**<0.001**
2-Heptanone, 4-methyl-	0	0	47,073 (52061)	**<0.001**
Benzaldehyde	0	0	133,970 (353300)	**<0.001**
Dimethyl trisulfide	0	0	144,442 (274141)	**<0.001**
Benzene, 1,2,4-trimethyl-	0	20,059 (15274)	0	**<0.001**
Eucalyptol	0	10,123 (10887)	0	**<0.001**
Benzeneacetaldehyde	0	11,595 (8828)	0	**<0.001**
Benzaldehyde, 4-methyl-	0	19,657 (26858)	37,934 (66294)	**<0.001**
Benzeneacetaldehyde, α-methyl-	28,315 (49689)	95,472 (108403)	115,515 (267576)	0.378
Nonanal	0	0	200,984 (310859)	**<0.001**
p-Mentha-1,5-dien-8-ol	0	5725 (12494)	3411 (7159)	**0.016**
Cyclohexanol, 5-methyl-2-(1-methylethyl)-	0	0	137,948 (361046)	**<0.001**
Pentanenitrile, 5-(methylthio)-	0	8892 (30567)	5338 (13114)	0.072
Benzaldehyde, 2,5-dimethyl-	0	75,705 (93371)	101,796 (141317)	**<0.001**
4-(2-Furyl) pyridine	0	0	37,285 (111075)	**<0.001**
Benzaldehyde, 4-propyl-	63,858 (21117)	110,480 (97065)	126,138 (140007)	0.953
1-Decanol	0	387,312 (424772)	0	**<0.001**
Benzenamine, 3,5-dichloro-	0	23,736 (37229)	32,300 (78139)	**0.003**
Propofol	0	0	28,265 (96039)	**0.026**
Benzene, (isothiocyanatomethyl)-	23,113 (39377)	0	0	**0.002**
2(3 H)-Furanone, 5-hexyldihydro-	0	123,646 (79831)	0	**<0.001**
1-Naphthalenecarboxaldehyde	4153 (9168)	0	0	**0.018**
Phenol, 2,5-bis(1,1-dimethylethyl)-	401,288 (160456)	905,390 (525181)	1,716,810 (188809)	**<0.001**
Benzoic acid, 4-ethoxy-, ethyl ester	0	3390 (4868)	15,596 (24557)	**<0.001**
Hexamethylene diacrylate	365,007 (187078)	240,518 (168062)	467,793 (618420)	0.288
2(3 H)-Furanone, dihydro-5-(2-octenyl)-, (Z)-	21,247 (16245)	29,239 (47547)	35,220 (56719)	0.962
Benzyl Benzoate	0	64,092 (152849)	209,976 (719202)	**0.010**
Caffeine	0	0	492,162 (616067)	**<0.001**
Lidocaine	0	110,275 (427094)	25,769 (101469)	0.444
Oxybenzone	0	0	9656 (42628)	**0.012**

IFTA: Interstitial fibrosis and tubular atrophy; SD: Standard deviation

*p-values were obtained via the Kruskal-Wallis Test

### Evaluating the separation of the groups of urinary volatile organic compounds by linear discriminant analysis and leave-one-out cross validation

**Fig. 1:**
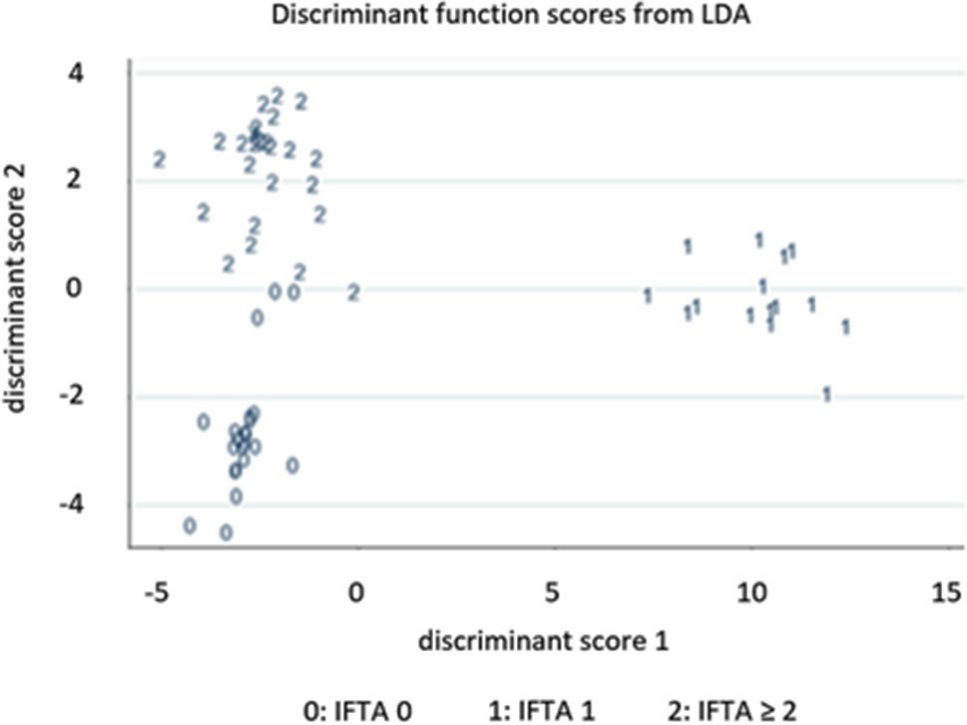
Linear discriminant analysis demonstrating individuals with IFTA 0, IFTA 1 and IFTA ≥ 2 could be significantly separated by their urinary VOCs profile. IFTA: Interstitial fibrosis and tubular atrophy

LDA results demonstrated three well-separated groups (i.e. individuals with IFTA 0, individuals with IFTA 1, and individuals with IFTA ≥ 2) (Fig. 1). This finding indicates the three IFTA groups are easily separable by their urinary VOC profile. LDA confirmed the pre-identified 34 urinary VOCs were statistically associated with the correct classification of study participants with IFTA 0, study participants with IFTA 1, or study participants with IFTA ≥ 2 (*p* < 0.001).

The Mahalanobis distance values were 176, 24, and 162 respectively between study participants with IFTA 0 and IFTA 1; between study participants with IFTA 0 and IFTA ≥ 2, and between study participants with IFTA 0 and IFTA ≥ 2 (all *p* < 0.001). Therefore, the current model displayed a very good discrimination of the three groups, particularly between individuals with IFTA 0 and IFTA 1, and between individuals with IFTA 1 and those with IFTA ≥ 2.

According to the LOO cross-validation results (Table 3), 86.4% of study participants (19 of 22 people) with IFTA 0 were classified correctly by their urinary VOCs profile; 86.7% of study participants (13 of 15 people) with IFTA 1 were classified correctly by their urinary VOCs profile; and 74.1% of study participants with IFTA ≥ 2 (20 of 27 people) were classified correctly by their urinary VOCs profile.

**Table 3:** Correct classification rate based on the LOO cross validation method

IFTA group	Number of study participants correctly classified
IFTA 0	86.4%
IFTA 1	86.7%
IFTA ≥ 2	74.1%

IFTA: Interstitial fibrosis and tubular atrophy; LOO: Leave-one-out

### Associations between individual urinary volatile organic compounds with IFTA status and covariates

Results from linear regression analysis evaluating associations between IFTA grading amongst the three study participant groups and expression levels of identified urinary VOCs are presented in Table 4. There were 5 VOCs from the univariate model and 4 VOCs from the multivariate model which were significantly upregulated in the IFTA 1 compared to the IFTA 0 group (*p* < 0.05), of which 2-heptanone; Benzene, 1,2,4-trimethyl; Benzeneacetaldehyde; and 2(3 H)-furanone, 5-hexyldihydro were significantly upregulated VOCs in both the univariate and multivariate analyses. There were 12 VOCs from the univariate model and 2 VOCs from the multivariate model which were significantly upregulated in the IFTA ≥ 2 compared to the IFTA 1 group (*p* < 0.05), of which 2-heptanone, 4-methyl and Benzaldehyde, 4-methyl were significantly upregulated VOCs in both the univariate and multivariate analyses. There are 2 VOCs (Benzene (isothiocyantomethyl) and Benzaldehyde, 2,5-dimethyl) in the univariate model which were positively associated with IFTA progression across all stages (*p* < 0.05), while no VOCs in the multivariate model displayed such statistical association.

**Table 4:** Associations between identified individual urinary volatile organic compounds and IFTA status & adjusted covariates as determined by linear regression analyses

	Univariate model							Multivariate model*							
	Coefficient		SE		95% CI		*p*-value	Coefficient			SE		95% CI		*p*-value
**Benzeneacetaldehyde**, α-**methyl**-															
**IFTA grading**															
IFTA 0	Reference							Reference							
IFTA 1	67,157		61,800		-56,420, 190,734		0.281	-25,149		95,284			-216,102, 165,804		0.793
IFTA ≥ 2	87,200		53,009		-18,798, 193,198		0.105	-87,971		117,774			-323,994, 148,053		0.458
**Age**								3149		1652			-162, 6460		0.062
**Sex**															
Female								Reference							
Male								-40,090		45,381			-131,036, 50,855		0.381
**eGFR**								-3046		1799			-6651, 559		0.096
**Diabetes presence**															
Without diabetes								Reference							
With diabetes								-231,251		88,653			-408,917, -53,586		**0.012**
**Albuminuria**															
No albuminuria								Reference							
Microalbuminuria								-30,061		79,520			-189,423, 129,301		0.707
Macroalbuminuria								91,230		81,913			-72,928, 255,388		0.270
**Benzaldehyde**,** 4-propyl-**															
**IFTA Grading**															
IFTA 0	Reference							Reference							
IFTA 1	46,621		34,589		-22,544, 115,787		0.183	52,778		54,059			-55,558, 161,114		0.333 0.777
IFTA ≥ 2	62,279		29,669		2952, 121,606		**0.040**	-19,056		68,818			-152,963, 114,850		
**Age**								577		937			-1302, 2455		0.541
**Sex**															
Female								Reference							
Male								-2476		25,747			-54,073, 49,122		0.924
**eGFR**								-2743		1021			-4788, -698		**0.009**
**Diabetes presence**															
Without diabetes								Reference							
With diabetes								-88,255		50,297			-189,053, 12,542		0.085
**Albuminuria**															
No albuminuria								Reference							
Microalbuminuria								-77,033		45,115			-167,446, 13,380		0.093
Macroalbuminuria								-43,751		46,473			-136,886, 49,383		0.351
**Phenol**,** 2**,**5-bis(1**,**1-dimethylethyl)-**															
**IFTA Grading**															
IFTA 0	Reference							Reference							
IFTA 1	504,102		422,592		-340,924, 1,349,128		0.238	91,153		686,206			-1,284,034, 1,466,341		0.895
IFTA ≥ 2	1,315,522		362,479		590,700, 2,040,344		**0.001**	390,564		848,173			-1,309,213, 2,090,341		0.647
**Age**								10,927		11,897			-12,914, 34,770		0.362
**Sex**															
Female								Reference							
Male								-405,889		326,822			-1,060,857, 249,078		0.220
**eGFR**								-19,421		12,954			-45,382, 6539		0.140
**Diabetes presence**															
Without diabetes								Reference							
With diabetes								-674,282		638,457			-1,953,779, 605,214		0.296
**Albuminuria**															
No albuminuria								Reference							
Microalbuminuria								-291,625		572,681			-1,439,305, 856,055		0.613
Macroalbuminuria								269,149		589,918			-913,073, 1,451,371		0.650
**2**,**3-Butanedione**															
**CKD presence**															
IFTA 0	Reference							Reference							
IFTA 1	-3.44e-11		105,892		-211,746, 211,746		1.000	-328,530		155,725			-640,611, -16,450		**0.039**
IFTA ≥ 2	255,634		90,829		74,009, 437,259		**0.007**	-150,833		192,481			-536,575, 234,909		0.437
**Age**								-418		2700			-5828, 4993		0.878
**Sex**															
Female								Reference							
Male								-91,657		74,168			-240,293, 56,979		0.222
**eGFR**								-5108		2939			-10,999, 783		0.088
**Diabetes presence**															
Without diabetes								Reference							
With diabetes								342,247		144,889			51,882, 632,612		**0.022**
**Albuminuria**															
No albuminuria								Reference							
Microalbuminuria								107,852		129,962			-152,598, 368,303		0.410
Macroalbuminuria								245,850		133,874			-22,439, 514,140		0.072
**4-Heptanone**															
**CKD presence**															
IFTA 0	Reference							Reference							
IFTA 1	-381,866		214,082		-809,949, 46,217		0.079	-363,642		336,295			-1,037,594, 310,309		0.284
IFTA ≥ 2	-869,378		183,628		-1,236,567, -502,189		**<0.001**	-478,159		415,672			-1,311,186, 354,867		0.255
**Age**								198		5830			-11,486, 11,882		0.973
**Sex**															
Female								Reference							
Male								-381,066		160,169			-702,052, -60,080		**0.021**
**eGFR**								10,035		6348			-2687, 22,757		0.120
**Diabetes presence**															
Without diabetes								Reference							
With diabetes								412,139		312,895			-214,916, 1,039,195		0.193
**Albuminuria**															
No albuminuria								Reference							
Microalbuminuria								107,627		280,659			-454,827, 670,082		0.703
Macroalbuminuria								144,927		289,107			-434,455, 724,311		0.618
**Styrene**															
**CKD presence**															
IFTA 0	Reference							Reference							
IFTA 1	53,014		70,379		-87,717, 193,746		0.454	-82,217		110,326			-303,317, 138,883		0.459
IFTA ≥ 2	132,886		60,367		12,173, 253,599		**0.032**	-23,865		136,367			-297,152, 249,421		0.862
**Age**								2931		1912			-901, 6765		0.131
**Sex**															
Female								Reference							
Male								-66,780		52,545			-172,085, 38,523		0.209
**eGFR**								-1895		2082			-6069, 2278		0.367
**Diabetes presence**															
Without diabetes								Reference							
With diabetes								-107,838		102,649			-313,553, 97,876		0.298
**Albuminuria**															
No albuminuria								Reference							
Microalbuminuria								-7446		92,074			-191,968, 177,075		0.936
Macroalbuminuria								155,481		94,845			-34,593, 345,557		0.107
**2-Heptanone**															
**IFTA Grading**															
IFTA 0	Reference							Reference							
IFTA 1	25,229		6555		12,120, 38,338		**<0.001**	34,885		10,630			13,582, 56,188		**0.002**
IFTA ≥ 2	-7.11e-12		5623		-11,244, 11,244		1.000	24,240		13,139			-2091, 50,571		0.070
**Age**								-101		184			-470, 268		0.585
**Sex**															
Female								Reference							
Male								-3589		5062			-13,735, 6556		0.481
**eGFR**								428		200			26, 830		**0.037**
**Diabetes presence**															
Without diabetes								Reference							
With diabetes								14,874		9890			-4946, 34,695		0.138
**Albuminuria**															
No albuminuria								Reference							
Microalbuminuria								-3423		8871			-21,202, 14,355		0.701
Macroalbuminuria								-3612		9138			-21,925, 14,701		0.694
**2-Heptanone**,** 4-methyl-**															
**IFTA Grading**															
IFTA 0	Reference							Reference							
IFTA 1	2.15e-11		11,381		-22,757, 22,757		1.000	11,497		18,491			-25,561, 48,555		0.537
IFTA ≥ 2	47,072		9762		27,552, 66,593		**<0.001**	57,787		22,856			11,981, 103,593		**0.014**
**Age**								-332		320			-974, 310		0.305
**Sex**															
Female								Reference							
Male								-4892		8807			-22,542, 12,757		0.581
**eGFR**								141		349			-557, 841		0.686
**Diabetes presence**															
Without diabetes								Reference							
With diabetes								-14,789		17,205			-49,269, 19,690		0.394
**Albuminuria**															
No albuminuria								Reference							
Microalbuminuria								9410		15,432			-21,517, 40,337		0.545
Macroalbuminuria								-6690		15,897			-38,549, 25,167		0.675
**Dimethyl trisulfide**															
**IFTA Grading**															
IFTA 0	Reference							Reference							
IFTA 1	3.44e-11		59,929		-119,836, 119,836		1.000	-40,474		99,361			-239,599, 158,650		0.685
IFTA ≥ 2	144,442		51,404		41,652, 247,231		**0.007**	102,216		122,814			-143,907, 348,341		0.409
**Age**								1574		1722			-1877, 5026		0.365
**Sex**															
Female								Reference							
Male								-45,838		47,323			-140,676, 48,999		0.337
**eGFR**								-400		1875			-4159, 3358		0.832
**Diabetes presence**															
Without diabetes								Reference							
With diabetes								-106,890		92,447			-292,159, 78,378		0.253
**Albuminuria**															
No albuminuria								Reference							
Microalbuminuria								-3593		82,923			-169,775, 162,589		0.966
Macroalbuminuria								66,709		85,419			-104,474, 237,893		0.438
**Benzene**,** 1**,**2**,**4-trimethyl-**															
**IFTA Grading**															
IFTA 0	Reference							Reference							
IFTA 1	20,058		2450		15,159, 24,958		**<0.001**	20,856		4005			12,829, 28,882		**<0.001**
IFTA ≥ 2	-5.93e-12		2101		-4202, 4202		1.000	705		4950			-9215, 10,626		0.887
**Age**								76		69			-62, 215		0.274
**Sex**															
Female								Reference							
Male								1042		1907			-2780, 4865		0.587
**eGFR**								8		75			-142, 160		0.908
**Diabetes presence**															
Without diabetes								Reference							
With diabetes								-8334		3726			-15,802, -866		**0.029**
**Albuminuria**															
No albuminuria								Reference							
Microalbuminuria								-1243		3342			-7942, 5455		0.711
Macroalbuminuria								608		3443			-6292, 7508		0.860
**Benzeneacetaldehyde**															
**IFTA Grading**															
IFTA 0	Reference							Reference							
IFTA 1	11,594		1416		8736, 14,426		**<0.001**	9902		2162			5569, 14,236		**<0.001**
IFTA ≥ 2	0		1214		-2428, 2428		1.000	2314		2672			-3041, 7670		0.390
**Age**								-14		37			-89, 60		0.696
**Sex**															
Female								Reference							
Male								1328		1029			-735, 3392		0.203
**eGFR**								93		40			11, 175		**0.026**
**Diabetes presence**															
Without diabetes								Reference							
With diabetes								4047		2011			15, 8079		**0.049**
**Albuminuria**															
No albuminuria								Reference							
Microalbuminuria								1594		1804			-2022, 5210		0.381
Macroalbuminuria								3813		1858			87, 7538		**0.045**
**Benzaldehyde**,** 4-methyl-**															
**IFTA Grading**															
IFTA 0	Reference							Reference							
IFTA 1	19,656		15,119		-10,576, 49,889		0.198	30,518		23,315			-16,207, 77,245		0.196
IFTA ≥ 2	37,934		12,968		12,001, 63,866		**0.005**	79,607		28,819			21,852, 137,362		**0.008**
**Age**								-372		404			-1182, 437		0.361
**Sex**															
Female								Reference							
Male								-2162		11,104			-24,417, 20,091		0.846
**eGFR**								1152		440			269, 2034		**0.011**
**Diabetes presence**															
Without diabetes								Reference							
With diabetes								14,104		21,693			-29,370, 57,579		0.518
**Albuminuria**															
No albuminuria								Reference							
Microalbuminuria								33,811		19,458			-5184, 72,807		0.088
Macroalbuminuria								5991		20,044			-34,178, 46,160		0.766
**Nonanal**															
**IFTA Grading**															
IFTA 0	Reference							Reference							
IFTA 1	0		67,956		-135,886, 135,886		1.000	-59,269		110,067			-279,848, 161,310		0.592
IFTA ≥ 2	200,983		58,289		84,426, 317,540		**0.001**	89,490		136,046			-183,153, 362,133		0.513
**Age**								1218		1908			-2606, 5042		0.526
**Sex**															
Female								Reference							
Male								-104,244		52,422			-209,301, 811		0.052
**eGFR**								-884		2077			-5048, 3279		0.672
**Diabetes presence**															
Without diabetes								Reference							
With diabetes								-94,279		102,408			-299,510, 110,950		0.361
**Albuminuria**															
No albuminuria								Reference							
Microalbuminuria								116,500		91,857			-67,586, 300,587		0.210
Macroalbuminuria								40,073		94,622			-149,553, 229,701		0.674
**Benzaldehyde**,** 2**,**5-dimethyl-**															
**IFTA Grading**															
IFTA 0	Reference							Reference							
IFTA 1	75,704		34,332		7052, 144,357		**0.031**	91,458		57,975			-24,726, 207,643		0.120
IFTA ≥ 2	101,795		29,448		42,909, 160,682		**0.001**	109,524		71,659			-34,083, 253,132		0.132
**Age**								-839		1005			-2853, 1174		0.407
**Sex**															
Female								Reference							
Male								-28,403		27,612			-83,739, 26,932		0.308
**eGFR**								-117		1094			-2310, 2076		0.915
**Diabetes presence**															
Without diabetes								Reference							
With diabetes								20,832		53,940			-87,268, 128,932		0.701
**Albuminuria**															
No albuminuria								Reference							
Microalbuminuria								7671		48,383			-89,291, 104,635		0.875
Macroalbuminuria								-19,789		49,840			-119,670, 80,092		0.693
**Benzenamine**,** 3**,**5-dichloro-**															
**IFTA Grading**															
IFTA 0	Reference							Reference							
IFTA 1	23,736		18,095		-12,448, 59,921		0.195	10,877		30,230			-49,706, 71,461		0.720
IFTA ≥ 2	32,300		15,521		1262, 63,337		**0.042**	-8275		37,366			-83,159, 66,607		0.826
**Age**								-82		524			-1133, 967		0.875
**Sex**															
Female								Reference							
Male								-11,715		14,398			-40,569, 17,139		0.419
**eGFR**								-750		570			-1893, 393		0.194
**Diabetes presence**															
Without diabetes								Reference							
With diabetes								-22,291		28,127			-78,659, 34,076		0.431
**Albuminuria**															
No albuminuria								Reference							
Microalbuminuria								14,028		25,229			-36,532, 64,589		0.580
Macroalbuminuria								2309		25,988			-49,773, 54,391		0.930
**Benzene**,** (isothiocyanatomethyl)-**															
**IFTA Grading**															
IFTA 0	Reference							Reference							
IFTA 1	-23,112		7736		-38,582, -7643		**0.004**	-23,152		12,176			-47,555, 1249		0.062
IFTA ≥ 2	-23,112		6635		-36,381, -9844		**0.001**	-19,550		15,050			-49,712, 10,612		0.199
**Age**								-53		211			-476, 369		0.802
**Sex**															
Female								Reference							
Male								18,667		5799			7045, 30,290		**0.002**
**eGFR**								82		229			-377, 543		0.719
**Diabetes presence**															
Without diabetes								Reference							
With diabetes								-2036		11,329			-24,741, 20,667		0.858
**Albuminuria**															
No albuminuria								Reference							
Microalbuminuria								-2488		10,162			-22,854, 17,876		0.807
Macroalbuminuria								3062		10,468			-17,915, 24,041		0.771
**2(3 H)-Furanone**,** 5-hexyldihydro-**															
**IFTA Grading**															
IFTA 0	Reference							Reference							
IFTA 1	123,646		12,806		98,038, 149,253		**<0.001**	131,455		18,911			93,556, 169,354		**<0.001**
IFTA ≥ 2	2.85e-11		10,984		-21,964, 21,964		1.000	13,709		23,375			-33,135, 60,554		0.560
**Age**								521		327			-135, 1178		0.117
**Sex**															
Female								Reference							
Male								10,037		9007			-8013, 28,087		0.270
**eGFR**								219		357			-496, 934		0.542
**Diabetes presence**															
Without diabetes								Reference							
With diabetes								46,048		17,595			10,785, 81,310		**0.011**
**Albuminuria**															
No albuminuria								Reference							
Microalbuminuria								-15,483		15,782			-47,112, 16,146		0.331
Macroalbuminuria								-26,354		16,257			-58,935, 6226		0.111
**1-Naphthalenecarboxaldehyde**															
**IFTA Grading**															
IFTA 0	Reference							Reference							
IFTA 1	-4153		1801		-7755, -551		**0.025**	-3900		2936			-9784, 1983		0.189
IFTA ≥ 2	-4153		1544		-7242, -1064		**0.009**	-3257		3629			-10,530, 4015		0.373
**Age**								-26		50			-128, 75		0.600
**Sex**															
Female								Reference							
Male								3333		1398			530, 6135		**0.021**
**eGFR**								13		55			-97, 124		0.807
**Diabetes presence**															
Without diabetes								Reference							
With diabetes								-54		2731			-5529, 5420		0.984
**Albuminuria**															
No albuminuria								Reference							
Microalbuminuria								-591		2450			-5502, 4318		0.810
Macroalbuminuria								384		2524			-4673, 5443		0.879
**Benzoic acid**,** 4-ethoxy-**,** ethyl ester**															
**IFTA Grading**															
IFTA 0	Reference							Reference							
IFTA 1	3389		5424		-7458, 14,237		0.534	4073		8811			-13,585, 21,733		0.646
IFTA ≥ 2	15,596		4653		6291, 24,900		**0.001**	21,213		10,891			-614, 43,040		0.057
**Age**								78		152			-227, 384		0.611
**Sex**															
Female								Reference							
Male								-8659		4196			-17,069, -248		**0.044**
**eGFR**								166		166			-167, 499		0.323
**Diabetes presence**															
Without diabetes								Reference							
With diabetes								-830		8198			-17,261, 15,600		0.920
**Albuminuria**															
No albuminuria								Reference							
Microalbuminuria								3681		7354			-11,056, 18,418		0.619
Macroalbuminuria								2277		7575			-12,903, 17,459		0.765
**Caffeine**															
**IFTA Grading**															
IFTA 0	Reference							Reference							
IFTA 1	1.37e-10		134,677		-269,303, 269,303		1.000	-156,089		218,784			-594,541, 282,363		0.479
IFTA ≥ 2	492,162		115,519		261,166, 723,157		**<0.001**	213,178		270,424			-328,763, 755,120		0.434
**Age**								3821		3793			-3779, 11,423		0.318
**N Sex**															
Female								Reference							
Male								-158,626		104,201			-367,450, 50,197		0.134
**eGFR**								-4065		4130			-12,342, 4211		0.329
**Diabetes presence**															
Without diabetes								Reference							
With diabetes								-320,246		203,560			-728,190, 87,697		0.121
**Albuminuria**															
No albuminuria								Reference							
Microalbuminuria								59,197		182,588			-306,718, 425,113		0.747
Macroalbuminuria								157,724		188,084			-219,204, 534,654		0.405

eGFR: Estimated glomerular filtration rate; IFTA: Interstitial fibrosis and tubular atrophy; SE: Standard error; 95%CI: 95% confidence intervals of the coefficient

*Adjusted for age, sex, the level of eGFR, diabetes mellitus status, and albuminuria status

On evaluating associations between identified urinary VOCs and adjusted covariates within the multivariate linear regression model (Table 4), there were 2 VOCs (4-Hepatanone; and Benzoic acid, 4-ethoxy, ethyl ester) which were downregulated and 2 VOCs (Benzene (isothiocyantomethyl); and 1-Napthalenecarboxaldeyde) which were upregulated with the male sex. There were 3 VOCs (Benzaldehyde, 4-propyl; 2-heptanone; and Benzaldehyde, 4-methyl) which were positively associated with decline in eGFR levels. There were 3 VOCs ( 2,3-butanedione; Benzeneacetaldehyde; and 2(3 H)-Furanone, 5-hexyldihydro) which were positive associated with DM status. Benzeneacetaldehyde was positively associated with albuminuria status.

## Discussion

This study is the first that has evaluated the associations between expression levels of urinary VOCs and kidney tubulointerstitial histopathology. It is particularly significant in a CKD context, given IFTA is the hallmark of CKD. Overall, our results identified 34 VOCs which enabled classification between individuals with no tubulointerstitial disease, mild tubulointerstitial disease and moderate/severe tubulointerstitial disease. Our multivariate regression analysis model evaluating the association between expression levels of urinary VOCs and CKD adjusted for age, sex, eGFR, diabetic and albuminuria status, given these covariates were determined to be significantly associated with VOCs expression and CKD progression from previous studies [19–21]. In the multivariate analysis, we identified 4 VOCs significantly upregulated in the mild IFTA compared to the no IFTA group and 2 VOCs significantly upregulated in the moderate/severe IFTA compared to the mild IFTA group.

Metabolic dysregulation that occurs with CKD progression is primarily characterized by oxidative stress and inflammation [22]. Increased production of reactive oxygen species (ROS) results in oxidative damage to lipids, proteins and DNA through their reactive properties [23]. Emerging evidence suggests ROS also function as important secondary messengers in cellular signalling pathways [24, 25]. For one, cytoplasmic ROS induces the activity of AMP-activated protein kinase, which has a crucial role in glucose and lipid metabolism, cell survival, growth, and inflammation, all of which are affected in CKD [24, 26]. Oxidative stress can also activate the transcription factor NF-κB, which induces the expression of cytokines and chemokines to regulate inflammatory responses in the kidneys [27]. The inflammatory cascade in CKD is characterized by the generation and/or accumulation of proinflammatory cytokines (e.g. tumour necrosis factor-α and interleukin-1) from intrinsic and/or extrinsic kidney damage not limited to uraemia, dyslipidaemia, malnutrition, infection and gut microbiota, resulting in increased blood flow, upregulation of chemical mediators and leukocyte infiltration [28]. Prior investigations established physiological links between VOCs and oxidative stress, lipid and amino acid metabolism, and inflammation [29, 30]. Hence, there is a basis in CKD for utilizing metabolomic markers such as VOCs to capture the extent of oxidative stress and inflammation, and translationally inform on the degree of CKD progression.

The majority of the 34 identified urinary VOCs in our study, and all of the significantly upregulated urinary VOCs belonged to one of four key organic chemistry functional groups - aldehydes, ketones, hydrocarbons, and alcohols. Urinary aldehydes can be exogenous or endogenous in origin. They can be produced during lipid peroxidation via the beta-cleavage reaction of lipid alkoxyl radicals [31]. It is well-known that there are lipid metabolic disturbances in patients with kidney disease [32]. Therefore, abnormal urinary aldehyde levels in these conditions may be explained by the lipid peroxidation damage that occurs. Ketones typically originate from exogenous sources and from the decarboxylation of oxo-acids [33, 34]. In healthy humans, ketones are mainly formed in hepatocytes from acetoacetate during the decarboxylation of excess acetyl-CoA [34]. Human breath, blood and urine all contain ketones in the form of acetone [34]. Heptanone in urine is supposedly the product of beta-oxidation of 2-ethylhexanoic acid, a metabolic product of the plasticizer di-(2-ethylhexyl)-phthalate [10]. Impairment of kidney function may reduce the filtration of ketones, leading to decreased concentration of ketones detected in the urine of kidney disease patients [35]. There is emerging evidence nevertheless, which observed increased urinary ketone (2-pentanone) levels in kidney disease aetiologies such as idiopathic membranous nephropathy (IMN) [36]. Further study is needed to delineate the intricacies that are linked between kidney pathology and ketone physiology. Hydrocarbons are thought to be the by-product of cholesterol biosynthesis [37, 38]. Change in levels of urinary VOCs stemming from the hydrocarbon group (i.e. benzaldehydes and carbonyl groups) in kidney disease may indicate disorders in tryptophan metabolism and alterations in pyruvate, glycine, serine, and threonine metabolisms, respectively [39]. Alcohols originate from aliphatic alcohol in human tissue fluids, and various processes formed from acetaldehyde metabolism or exogenous intake [40]. Its role in oxidative stress and inflammation pathways in kidney disease is well-established [41].

Although there were no previous studies which evaluated associations between expression of urinary VOCs and CKD as defined by tubulointerstitial pathology, urinary VOCs have been previously studied for their potential as biomarkers in multiple glomerular diseases such as mesangial proliferative glomerulonephritis, Immunoglobulin A nephropathy, IMN and minimal change disease [36, 42–44]. In the preliminary studies that were conducted, a different panel of significantly upregulated (or downregulated) VOCs with progressing disease severity were identified, in comparison to the identified VOCs from our study [36, 42–44]. Wang et al. [42] evaluated urine samples in 15 mesangial proliferative glomerulonephritis (MPGN) patients, 21 Immunoglobulin A nephropathy (IgAN) patients and 15 healthy controls. Five VOCs (Carbamic acid, monoammonium salt; Carbon disulfide; Silanediol, dimethyl-; 2 H-1,4-Benzodiazepin-2-one, 7-chloro-1,3-dihydro-5-phenyl-1-(trimethylsilyl)- and Butylated Hydroxytoluene) had significantly elevated expression levels in the MPGN group compared with the control group, whilst 3 VOCs (Carbamic acid, monoammonium salt; Carbon disulfide and 2 H-1,4-Benzodiazepin-2-one,7-chloro-1,3-dihydro-5-phenyl-1-(trimethylsilyl)-) were found at increased expression levels in the IgAN group compared to normal controls. In addition, 5 VOCs (Tartronic acid; Carbamic acid; Sulfide, allyl methyl; Hydrogen azide and Benzeneethanamine, N-[(pentafluorophenyl)methylene]-.beta,4-bis[(trimethylsilyl)oxy]-) were significantly increased in IgAN patients compared with MPGN patients, suggesting these urinary VOCs may be specific biomarkers which differentiate between the two conditions. 4-heptanone, 2-pentanone and pyrrole were identified at decreased urinary levels in IgAN and MPGN patients compared to the control groups. Wang et al. [43] also aimed to detect urinary VOCs which could distinguish between patients with idiopathic membranous nephropathy (IMN) and normal controls. The investigators assessed the urine collected from 63 IMN patients and 15 normal controls, in which 6 VOCs (Carbamic acid, monoammonium salt; 2-pentanone; 2,4-dimethyl-pentanal; Hydrogen azide; Thiourea and 4-heptanone) displayed significantly higher expression levels in IMN patients compared to normal controls. The same investigator group [36] also collected urine samples from 38 minimal change disease (MCD) patients and 15 healthy controls. They identified 6 VOCs (Trans-2,2-dimethyl-4-decene; Pyrrole; Carbamic acid, monoammonium salt; 1-butyne, 3,3-dimethyl-; Diisopropylamine and 4-heptanone) that are present at reduced urinary expression levels in MCD patients. Further work is needed to validate the use of these urinary VOCs as biomarkers to predict MCD status and disease progression. A more recently conducted study by Ligor et al. [44], which separated and identified urinary VOCs via gas chromatography time-of-flight mass spectrometry, aimed to determine urinary VOC profiles between 27 patients diagnosed with glomerular diseases and 20 healthy controls. Amongst those diagnosed with glomerular disease, there were 4 VOCs (Methyl hexadecanoate; 9-hexadecen-1-ol; 6,10-dimethyl-5,9-undecadien-2-one and 2-pentanone) found to be at elevated urinary expression levels.

Otherwise, links between exhaled air VOCs from human breath with CKD were recently investigated. Romani et al. [45] examined the utility of selected ion flow tube-mass spectrometry (SIFT-MS) to measure breath VOCs in CKD patients and healthy subjects, and evaluated the possible correlation between breath VOC expression levels with the presence of CKD and CKD progression as determined by the Kidney Disease Improving Global Outcomes guideline diagnostic criteria [46]. The investigators enrolled 68 Stage I-IV CKD patients (all were receiving conservative therapy) and 54 healthy subjects. Analysis of the VOCs from exhaled air of the enrolled subjects was performed by SIFT-MS. They observed increased breath VOCs expression levels for numerous VOCs in CKD compared to healthy subjects and with progressing CKD severity, albeit these were different VOCs from the ones identified in our study. The most relevant results by receiver operating characteristic curves were observed for trimethylamine (TMA), acetone, ammonia, and dimethyl sulfide. Romani et al. [45] noted that an individual’s breath TMA concentration superior to 26 parts per billion by volume characterizes a 6.11 times greater risk of having CKD, compared to those with lower levels of breath TMA concentration. Moreover, they detected an increased concentration of acetone and ammonia in CKD patients compared to healthy subjects. SIFT-MS is considered a superior mass spectrometry option for measuring nitrogen- and sulfur-containing VOCs, which are more challenging to measure when using other mass spectrometry modalities. Future studies evaluating urinary VOCs within a CKD context using SIFT-MS is anticipated.

Whilst our study findings provide novel evidence into the associations between urinary VOCs and CKD, there remain important gaps in our knowledge base which require evaluation. For one, the exact mechanisms for the generation of most urinary VOCs is unclear at a molecular level, and they could be perturbed in many physiological and pathological states outside of tubulointerstitial disease alone, Although we adjusted for several potential confounding factors in our analyses, there may be other factors challenging to control, not limited to dietary habits, physical stress and environmental exposure to toxins, which could affect the accuracy of urinary VOCs profiling [47]. Hence, further studies with larger clinical cohorts are required to validate our data, adjusting for other potential covariates that may be relevant to kidney disease. Another issue relates to the vast quantity of urinary VOCs that were found to be potentially useful biomarkers of CKD across different IFTA stages, also considering there may be other clinically significant urinary VOCs that remain unidentified currently. Further evidence to specify and narrow towards the key urinary VOCs that could be confidently applied in clinical practice to predict CKD progression is required. While most urinary VOCs and other metabolomic studies reported to date used GC-MS as the analytical method, complementary analysis could be performed by reversed-phase liquid chromatography-mass spectrometry (RP-LC-MS), hydrophilic interaction liquid chromatography-mass spectrometry (HILIC-LC-MS), and capillary electrophoresis-mass spectrometry (CE-MS) methods as well [48]. This would broaden the range of potential disease markers that could be investigated. Alternative types of mass spectrometry analysis approaches could also be considered to improve sensitivity of metabolite detection but this must be balanced against their increasing price, operating costs and complicated operation in a clinical setting [49]. Hence, improving biosensing software platforms to detect clinically useful urinary VOCs is an attractive proposition where ongoing technological developments are foreseeable. For one, the feasibility of metal oxide biosensor platforms to determine urinary VOCs with significant predictive capability for detecting genitourinary cancers (i.e. renal cell carcinoma, transitional cell carcinoma and prostate cancer) has been recently demonstrated to good levels of accuracy. Future studies could perhaps consider extending its use for this purpose in CKD [50]. Furthermore, a mass spectrometry-based electronic nose (MS-EN) approach possesses tremendous potential but has been seldomly applied for urinary VOCs and so far, has not been explored within in CKD yet though it has been trialled within the context of kidney cancer [51, 52]. This is also a potential avenue of further research to be considered.

## Conclusions

Our study demonstrated that the urinary expression levels of various aldehydes, ketones, hydrocarbons and alcohols are significantly associated with tubulointerstitial histopathology, which suggests urinary VOCs may indeed have a clinically useful role in CKD as a metabolomic biomarker. Additional studies are required to validate our findings in a larger cohort and examine the potential of utilizing urinary VOCs to reliably assess CKD progression in clinical practice.

## Data Availability

The datasets used and/or analysed during the current study are available from the corresponding author on reasonable request.

## Abbreviations

ANOVA: Analysis of Variance
CKD: Chronic Kidney Disease
DM: Diabetes Mellitus
eGFR: Estimated Glomerular Filtration Rate
GC: Gas Chromatography
GC-MS: Gas Chromatography–Mass Spectrometry
IFTA: Interstitial Fibrosis and Tubular Atrophy
IMN: Idiopathic Membranous Nephropathy
IS: Internal Standard
LDA: Linear Discriminant Analysis
LOO: Leave-one-out
MS-EN: Mass Spectrometry-based Electronic Nose
PTV: Programmed Temperature Vaporization
QC: Quality Control
ROS: Reactive Oxygen Species
SBSE: Stir Bar Sorptive Extraction
SIFT-MS: Selected Ion Flow Tube-Mass Spectrometry
VOCs: Volatile Organic Compounds
TD: Thermal Desorption
TDU: Thermal Desorption Unit
TMA: Trimethylamine
95%CI: 95% Confidence Intervals
